# Continuous monitoring of plant sodium transport dynamics using clinical PET

**DOI:** 10.1186/s13007-021-00707-8

**Published:** 2021-01-19

**Authors:** Gihan P. Ruwanpathirana, Darren C. Plett, Robert C. Williams, Catherine E. Davey, Leigh A. Johnston, Herbert J. Kronzucker

**Affiliations:** 1 Melbourne Brain Centre Imaging Unit, The University of Melbourne, Melbourne, VIC Australia; 2 Department of Biomedical Engineering, The University of Melbourne, Melbourne, VIC Australia; 3 Australian Plant Phenomics Facility, The Plant Accelerator, School of Agriculture, Food & Wine, University of Adelaide, Urrbrae, SA Australia; 4 Faculty of Veterinary and Agriculture Sciences, School of Agriculture and Food, The University of Melbourne, Melbourne, VIC Australia; 5 Faculty of Land and Food Systems, University of British Columbia, Vancouver, BC V6T 1Z4 Canada

**Keywords:** Positron emission tomography, Barley, ^22^Na transport dynamics, Inhibitor effect, Nutrient effect, Diurnal transport

## Abstract

**Background:**

The absorption, translocation, accumulation and excretion of substances are fundamental processes in all organisms including plants, and have been successfully studied using radiotracers labelled with ^11^C, ^13^N, ^14^C and ^22^Na since 1939. Sodium is one of the most damaging ions to the growth and productivity of crops. Due to the significance of understanding sodium transport in plants, a significant number of studies have been carried out to examine sodium influx, compartmentation, and efflux using ^22^Na- or ^24^Na-labeled salts. Notably, however, most of these studies employed destructive methods, which has limited our understanding of sodium flux and distribution characteristics in real time, in live plants. Positron emission tomography (PET) has been used successfully in medical research and diagnosis for decades. Due to its ability to visualise and assess physiological and metabolic function, PET imaging has also begun to be employed in plant research. Here, we report the use of a clinical PET scanner with a ^22^Na tracer to examine ^22^Na-influx dynamics in barley plants (*Hordeum vulgare* L. spp.* Vulgare*—cultivar Bass) under variable nutrient levels, alterations in the day/night light cycle, and the presence of sodium channel inhibitors.

**Results:**

3D dynamic PET images of whole plants show readily visible ^22^Na translocation from roots to shoots in each examined plant, with rates influenced by both nutrient status and channel inhibition. PET images show that plants cultivated in low-nutrient media transport more ^22^Na than plants cultivated in high-nutrient media, and that ^22^Na uptake is suppressed in the presence of a cation-channel inhibitor. A distinct diurnal pattern of ^22^Na influx was discernible in curves displaying rates of change of relative radioactivity. Plants were found to absorb more ^22^Na during the light period, and anticipate the change in the light/dark cycle by adjusting the sodium influx rate downward in the dark period, an effect not previously described experimentally.

**Conclusions:**

We demonstrate the utility of clinical PET/CT scanners for real-time monitoring of the temporal dynamics of sodium transport in plants. The effects of nutrient deprivation and of ion channel inhibition on sodium influx into barley plants are shown in two proof-of-concept experiments, along with the first-ever 3D-imaging of the light and dark sodium uptake cycles in plants. This method carries significant potential for plant biology research and, in particular, in the context of genetic and treatment effects on sodium acquisition and toxicity in plants.

## Background

The absorption, translocation, accumulation and excretion of substances are fundamental processes in plant physiology that have been successfully studied using radiotracer technologies, for their inherent sensitivity and quantitative accuracy [1–4]. These include methods that employ short-lived radiotracers, labelled with ^11^C and ^13^N, for real-time tracing, and long-lived radiotracers, labeled with ^14^C or ^22^Na, for measuring the accumulation of substances [5].

Nearly 80 years ago, Ruben et al. [6] conducted the first plant study using a short-lived radioisotope, ^11^C, to examine photosynthesis. Since then, many studies have been carried out using ^11^C to study photosynthesis and the carbohydrate source-sink relationship in plants. In addition to ^11^C, radioisotope ^13^N is used to study nutrient absorption and transportation characteristics under various conditions [7, 8].

One of the most damaging ions to crop growth and productivity, at levels encountered in agricultural soils, is sodium [9, 10]. Due to the significance of understanding sodium influx characteristics, several studies have been conducted using ^22^Na radioisotope [11–13], with some also employing ^24^Na to study both influx and efflux processes [14–16]. Nevo et al. [17] studied the variation of ^22^Na uptake in wild Emmer wheat in the context of salt tolerance. Smitharani et al. [18] showed that ^22^Na uptake is significantly lower in salt-tolerant groundnut varieties than in salt-sensitive ones. Notably, however, these studies employed destructive methods to determine radioactivity in plant organs after plant harvesting, not permitting the real-time monitoring of tracer distribution in the living plant.

With the development of high-throughput phenotyping platforms, non-destructive imaging techniques, including visual imaging, thermal infrared imaging, fluorescence imaging, and tomographic imaging, are being employed to analyse plants traits related to the growth, yield and adaptation to biotic and abiotic stress [19]. De Vylder et al. [20] developed a visible light image analysis platform to evaluate plant-shoot phenotypes, while Zia et al. [21] used infrared thermal imaging to identify water-stress tolerant maize genotypes. Windt et al. [22] used a dedicated Magnetic Resonance Imaging (MRI) scanner to compare phloem and xylem flow characteristics and dynamics in poplar, castor bean, tomato, and tobacco. Perret et al. [23] developed a protocol to visualize and quantify roots using computed tomography (CT) and tested it using chickpea plants.

Positron emission tomography (PET) imaging is a non-invasive imaging modality that records radiotracer pathways. It has been successfully used for decades by medical diagnostic and pharmaceutical industries due to its ability to assess physiological and metabolic function in the living, intact organism in real time. McKay et al. [24] were the first to describe the use of PET systems in plant research, studying the long-distance transport in plants by monitoring the movement of ^18^F through a separated soybean shoot. Since then, PET systems have been in sporadic use in plant research, to quantitatively assess the uptake, inter-organ transport, and storage of radio-labeled substances [25]. Recently, Converse et al. [2] used an animal PET imaging system to model and experimentally evaluate the uptake of atmospheric fluoride in plants by administrating ^18^F to the petioles of *Brassica oleracea*. De Schepper et al. [26] and Thorpe et al. [27] used ^11^C-labeled PET radiotracers to study ^11^C transport dynamics in oak phloem tissue and the long-distance transport of the phytohormone methyl jasmonate in tobacco plants, respectively. Recognising the importance of PET in plant research, a dedicated plant PET system, PlanTIS, was developed by Beer et al. [28]. Jahnke et al. [29] used PlanTIS to investigate the allocation of carbon to various plant organs by administering a ^11^C-labeled tracer into beet, radish, and maize plants. Streun et al. [30] developed a second plant-dedicated PET system, phenoPET, but its practical usage remains a work in progress.

There has been one study to date demonstrating the feasibility of clinical PET scanners for plant research, that of Karve et al. [31], in which a Siemens HR + PET scanner was used to quantitatively study C-photoassimilate transport dynamics and allocation patterns in sorghum plants, across vegetative and reproductive stages, by administrating ^11^CO_2_ to the plants. Although they optimised the scanner settings, 3-D data acquisition and attenuation and scatter correction using transmission scans, to maximise the quantitative accuracy, these scanner corrections, attenuation, scatter and decay corrections, are less accurate compared to correction methods such as CT based attenuation corrections in new clinical PET scanners.

There have been two studies to date of sodium influx in intact plants using PET imaging with a ^22^Na-labeled tracer. Fujimaki et al. [32] determined the translocation directions and rates of sodium ion in a salt-tolerant plant, common reed, and a salt-sensitive plant, rice, under high salt conditions using a positron-emitting tracer planar imaging system. They found absorption of ^22^Na in the common reed roots, but no transportation to the shoot, and detected ^22^Na movement from the basal part of the roots to the distal part in the chase step. In contrast, ^22^Na absorbed in rice was continuously transported and accumulated in the shoot. This study was limited by the planar PET system with limited angular resolution, providing only 2D images. Further, plants were exposed to a continuous light source, thus effects of photosynthetic activity and transpiration on sodium influx could not be accounted for, and, as well, the initial sodium influx of the experimental plants was not observed. Recently, Ariño-Estrada et al. [33] studied the potential of using a small animal PET scanner to understand different sodium-tolerant properties in plant varieties by quantitatively differentiating the sodium transport dynamics between them. In this experiment, 24 green foxtail plants, 12 plants from each of two genotypes, were incubated in a ^22^Na^+^-containing radioactive growth medium for 14 days and scanned at five time points. Consistent transport dynamics were observed within plants of the same variety and differences between genotypes, which proved the potential of preclinical PET scanners in high-throughput phenotyping of sodium ion transport. Although the initial sodium influx was determined, temporal dynamics were not acquired continuously, nor were diurnal changes of sodium influx examined.

In contrast to the previous studies utilizing animal PET and specialized plant PET scanners, we here demonstrate the utility of clinical PET/CT for plant imaging, by scanning plants continuously for up to 3 days. To the best of our knowledge, this is the first demonstration of imaging plants using a clinical PET scanner with CT based attenuation correction, which is advantageous for better radioactivity quantification in studying plant physiology and CT-based delineation of plant regions of interest (ROIs). In comparison to small PET scanners and specialised plant PET scanners, the large field-of-view (FOV) of clinical PET/CT scanners is beneficial in studying ion dynamics in larger plants. Our work demonstrates the sensitivity of PET-measured ^22^Na-uptake dynamics in plants when sodium influx rates are modified under variable nutrient provisions and the addition of ion channel inhibitors, while providing day/night light cycles consistent with the growth protocols of plants and maintained for the duration of the PET experiments.

## Results

**Fig. 1:**
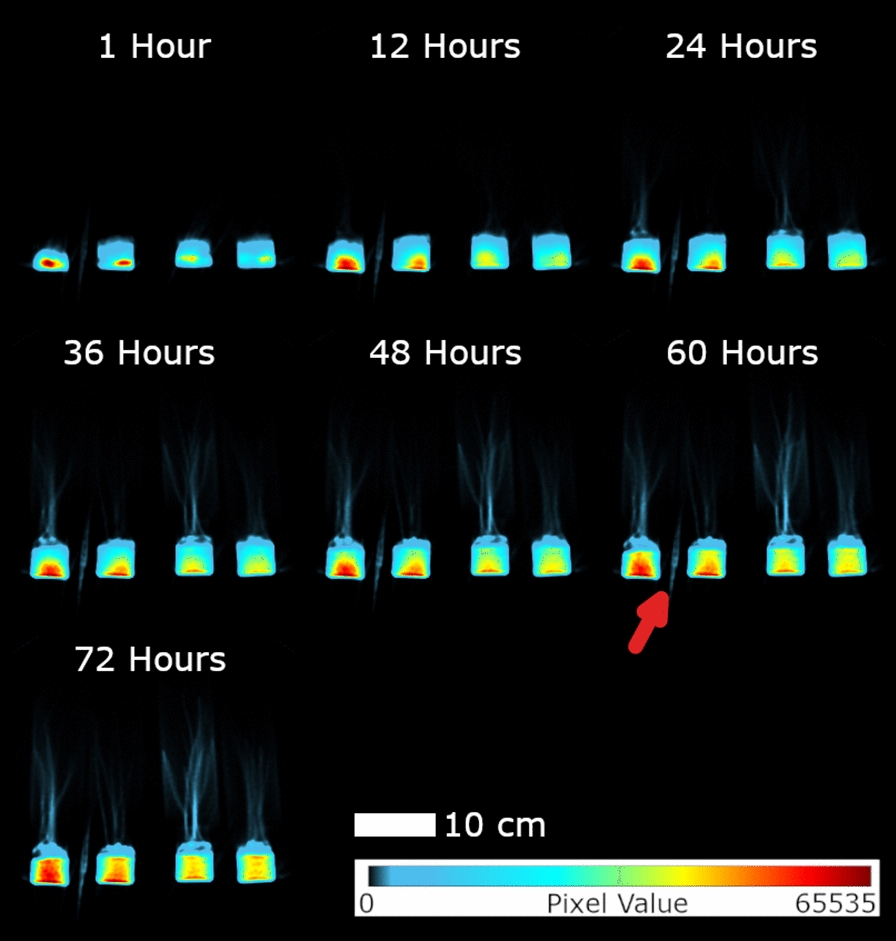
Dynamic images of Experiment 2 to capture ^22^Na movement in linear scale. Images were scaled identically. Red arrow shows the reference source. At each time point, Voxel values of slices containing plants were summed to generate these images. Voxel size is 1.59 mm × 1.59 mm. In each timeframe, from left to right: high nutrient plants grouping without BaCl_2_, high nutrient plants grouping with BaCl_2_, low nutrient plants grouping without BaCl_2_ and low nutrient grouping with BaCl_2_

**Fig. 2:**
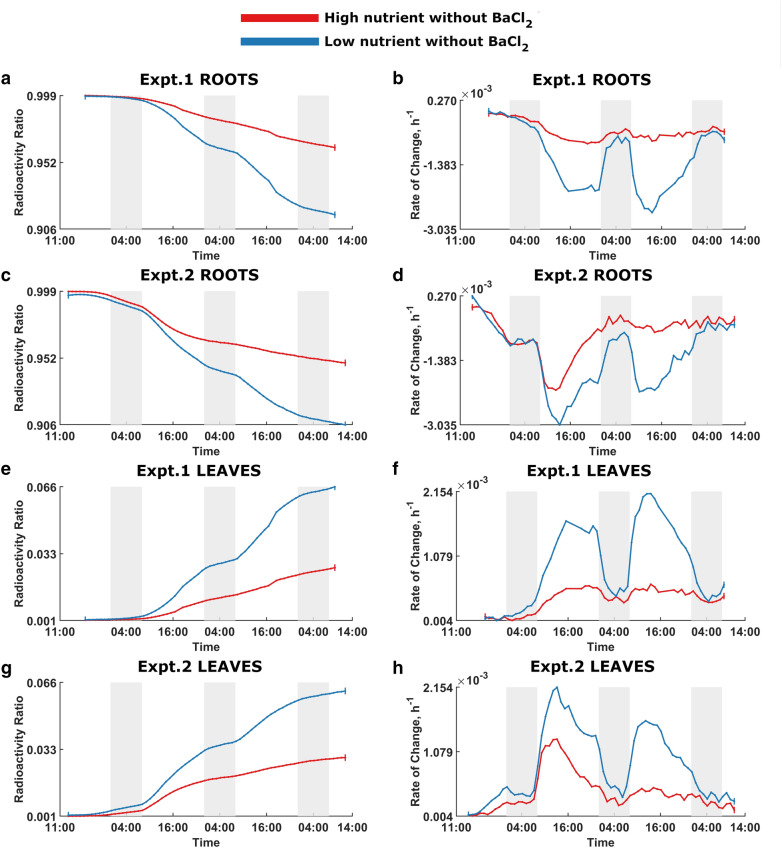
Nutrient effect on ^22^Na uptake in plants without BaCl_2_. The red and blue curves denote high-nutrient and low-nutrient plants without BaCl_2_, respectively. Dark shading indicates night times. Expt.1 Roots: **a** Radioactivity ratio. **b** Rate of change of the radioactivity ratio. Expt.2 Roots: **c** Radioactivity ratio. **d** Rate of change of the radioactivity ratio. Expt.1 Leaves: **e** Radioactivity ratio. **f** Rate of change of the radioactivity ratio. Expt.2 Leaves:** g** Radioactivity ratio. **h** Rate of change of the radioactivity ratio

**Fig. 3:**
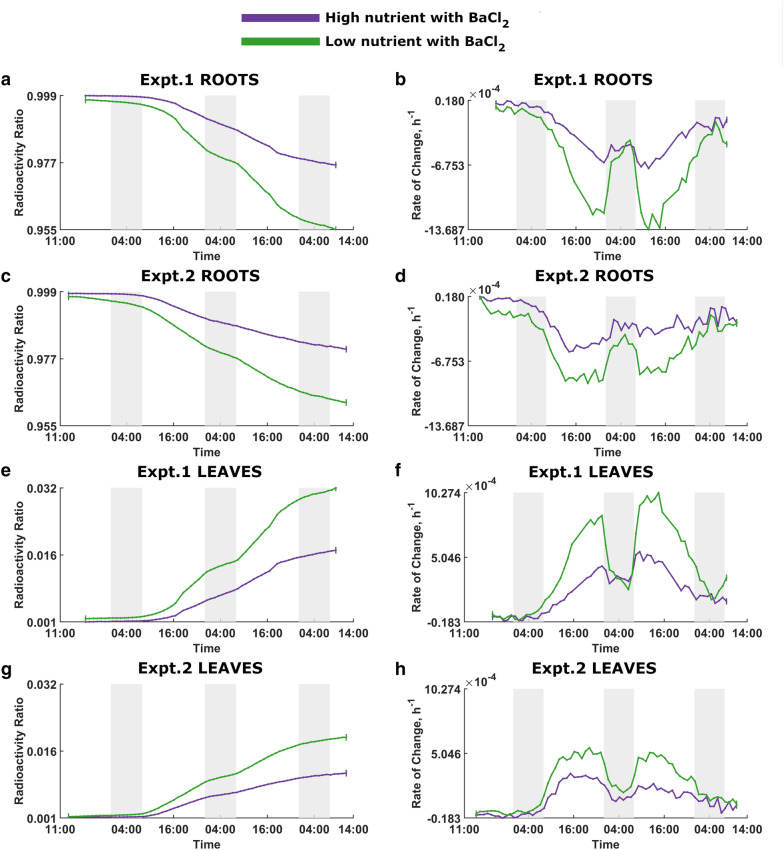
Nutrient effect on ^22^Na uptake in plants with BaCl_2_. The purple and green curves denote high-nutrient and low-nutrient plants with BaCl_2_, respectively. Dark shading indicates night times. Expt.1 Roots:** a** Radioactivity ratio. **b** Rate of change of the radioactivity ratio. Expt.2 Roots: **c** Radioactivity ratio. **d** Rate of change of the radioactivity ratio. Expt.1 Leaves: **e** Radioactivity ratio. **f** Rate of change of the radioactivity ratio. Expt.2 Leaves: **g** Radioactivity ratio. **h** Rate of change of the radioactivity ratio

**Fig. 4:**
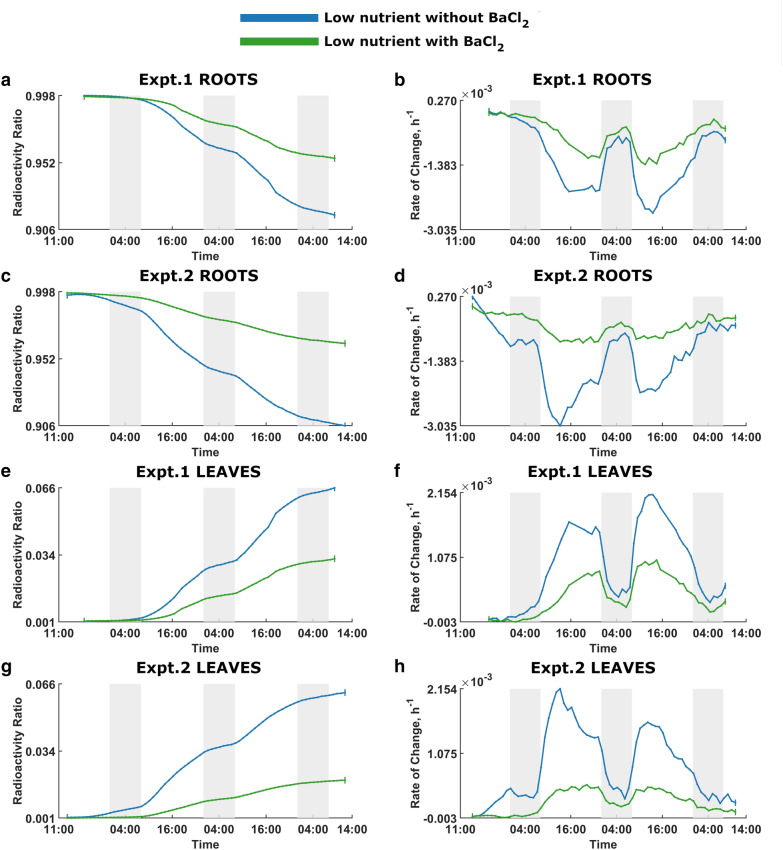
BaCl_2_ effect on ^22^Na uptake in low-nutrient plants. The green and blue curves denote low-nutrient plants with and without BaCl_2_, respectively. Dark shading indicates night times. Expt.1 Roots:** a** Radioactivity ratio. **b** Rate of change of the radioactivity ratio. Expt.2 Roots: **c** Radioactivity ratio. **d** Rate of change of the radioactivity ratio. Expt.1 Leaves: **e** Radioactivity ratio. **f** Rate of change of the radioactivity ratio. Expt.2 Leaves: **g** Radioactivity ratio. **h** Rate of change of the radioactivity ratio

**Fig. 5:**
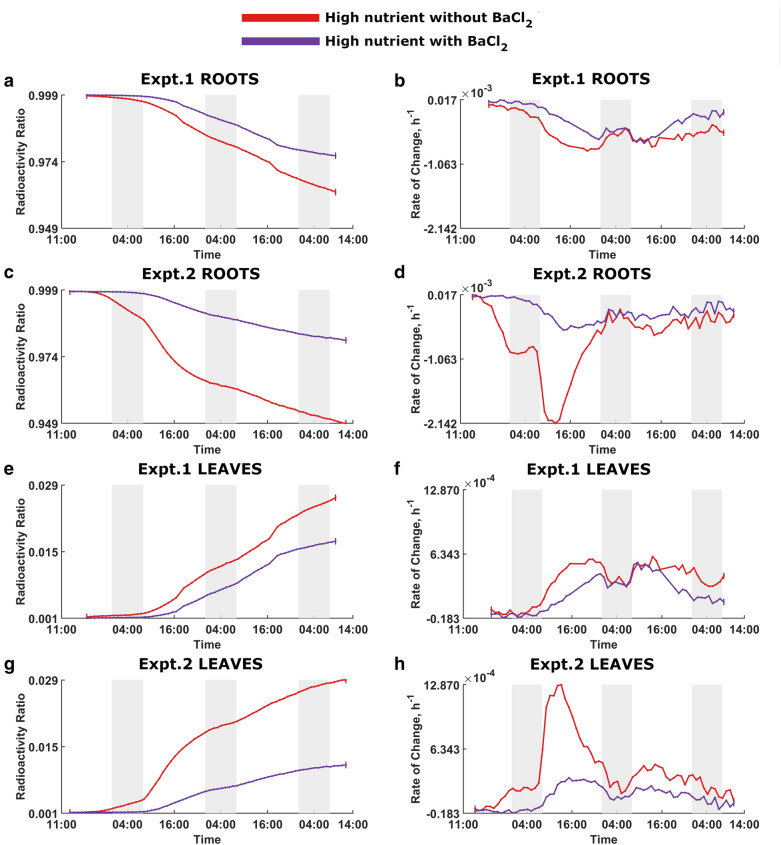
BaCl_2_ effect on ^22^Na uptake in high-nutrient plants. The purple and red curves denote high-nutrient plants with and without BaCl_2_, respectively. Dark shading indicates night times. Expt.1 Roots:** a** Radioactivity ratio. **b** Rate of change of the radioactivity ratio. Expt.2 Roots: **c** Radioactivity ratio. **d** Rate of change of the radioactivity ratio. Expt.1 Leaves:** e** Radioactivity ratio. **f** Rate of change of the radioactivity ratio. Expt.2 Leaves: **g** Radioactivity ratio. **h** Rate of change of the radioactivity ratio

Dynamic PET images of the whole plants, for example from Experiment 2 (Fig. 1), indicated clear ^22^Na uptake and translocation from the roots to the shoots in each plant grouping with levels influenced both by nutrient status and the presence/absence of BaCl_2_. Time-lapse 3D-image sequences of radioactivity dynamics for both experiments are provided in Additional files 1 and 2. The analysis of activity over time in each plant condition was remarkably consistent between the experiments for both leaves and roots (Figs. 2, 3, 4 and 5), showing the robustness of the experimental protocol. Due to the influx of ^22^Na, the relative radioactivity in the roots decreased while it increased in the leaves.

Figure 2 shows the nutrient effect on ^22^Na uptake in plants without BaCl_2_ and, as expected, low-nutrient plants absorb ^22^Na at higher rates than the high-nutrient plants. Figure 3 captures the dynamic behaviour of ^22^Na uptake in plants with BaCl_2_, demonstrating that low-nutrient plants transport more ^22^Na than high-nutrient plants.

Figures 4 and 5, which are rearrangements of Figs. 2 and 3, illustrate the effect of BaCl_2_ on ^22^Na uptake in low-nutrient and high-nutrients plants, respectively. In both instances, plants without BaCl_2_ show greater ^22^Na uptake.

As per the figures, there is no significant difference between two comparison plant groups at the beginning of the experiments; with time, the difference become noticeable, and treatments clearly diverge over time. Moreover, there is a discernible diurnal pattern of ^22^Na influx in all rate of change of relative radioactivity curves where plants absorb significantly more ^22^Na during the light period, and appear to anticipate the change in light provision by adjusting the ^22^Na-uptake rate to a significantly decreased rate in the dark.

**Fig. 6:**
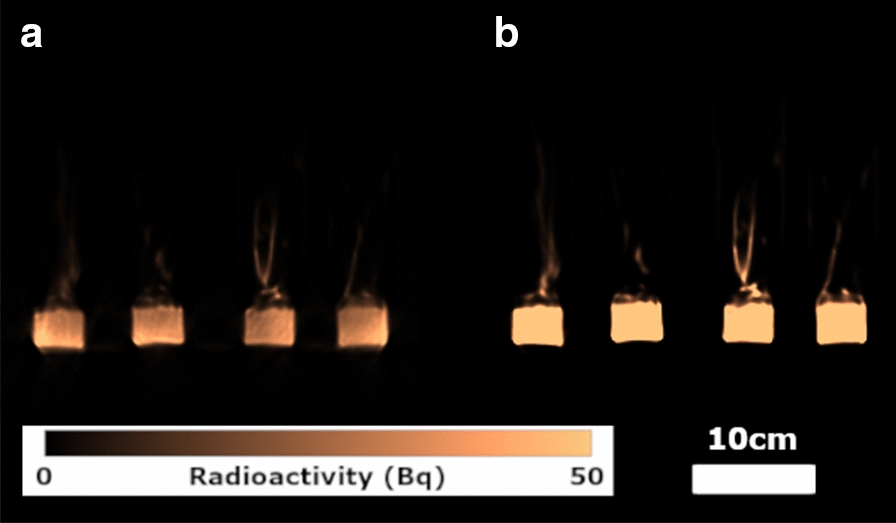
Comparison of PET images reconstructed **a** without and **b** with CT-based Attenuation Correction. The images without correction contain significant inaccuracies in the voxel localized radioactivity measures

At the end of Experiment 1, radioactivity values in each plant grouping, after the transportation of ^22^Na to the shoot, were calculated using PET images with and without CT based Attenuation Correction. Since ^22^Na is a radioisotope with a half-life of 2.605 years, true radioactivity in each plant grouping will be approximately equal to the administered dose, that we consider the ground truth total radioactivity values. Table 1 shows that radioactivity values estimated using CT-based Attenuation Correction of the raw PET data are consistently slight overestimates of ground truth. In contrast, radioactivity values were strikingly underestimated without CT-base Attenuation Correction. Figure 6 provides an exemplar image slice reconstructed with and without Attenuation Correction, clearly demonstrating its utility.

**Table 1:** Comparison of estimated total PET radioactivity with and without CT-based Attenuation Correction with the ground truth radioactivity values in Experiment 1

	High nutrient without inhibitor radioactivity (MBq)	High nutrient with inhibitor radioactivity (MBq)	Low nutrient without inhibitor radioactivity (MBq)	Low nutrient with inhibitor radioactivity (MBq)
Ground truth	1.74	1.79	1.69	1.70
Estimated with CT Attenuation Correction	1.88	1.88	1.78	1.78
Estimated without CT Attenuation Correction	0.81	0.72	0.72	0.76

Note that radioactivity values include the beaker and solution in which the plants are situated during the experiments

## Discussion

In this study, we analysed sodium influx dynamics in barley plants using a clinical PET/CT scanner. Past studies on plants have primarily been carried out on specialised plant PET scanners or small animal PET scanners, with the exception of Karve et al. [31]. Plant or small animal PET scanners have small fields-of-view, which limit the number of plants that can be simultaneously scanned, and the size of the plants. In contrast, clinical PET scanners have larger FOVs and controllable bed positioning are advantageous for scanning full-sized plants and multiple plants at once, as in the four scanned simultaneously in the current experiments. This demonstrates the potential for clinical PET systems in high-throughput phenotyping of ion transport, furthermore critical as salinity transport and tolerance traits can be uniquely expressed at different developmental stages in crop plants [34, 35].

Moreover, dedicated plant PET scanners are not accessible to most plant research groups, while clinical PET scanners exist in many research institutions. Clinical PET scanners provide high-level correction methods in image reconstruction to maximise the radioactivity quantification accuracy, higher spatial resolution and higher sensitivity, which are all advantageous in the accurate quantification of ion distribution in plants. As demonstrated by the estimation of total radioactivity (Table 1) and voxelwise localised intensities (Fig. 6), CT-based Attenuation Correction, a crucial element of every in vivo PET scan, is equally important in plant imaging for obtaining accurate radioactivity estimates. The use of CT imaging in plant studies has prompted studies on the effects of radiation exposure [36, 37]. It is important to distinguish between clinical (human) PET/CT systems, and µCT systems. Experiments such as the ones we report on clinical PET/CT scanners provide approximately the same radiation exposure as plants would absorb in 1–2 days of background radiation outdoors. The exposure from µCT systems, in contrast, is 6–7 orders of magnitude higher.

In previous studies, PET images or plant setup photographs, co-registered with PET images were used to acquire the plant’s structural information to mark ROIs for analysis. Due to thin tissues in plants, plant PET images are more susceptible to partial volume effects and it is challenging to mark ROIs on these images [38]. We exploited the CT component of the clinical PET/CT scanner to mark ROIs on PET images. Therefore, beyond the superior attenuation correction using a PET/CT system, they are advantageous in delineating small plant structures for ROI analyses.

In this study, we included a low-nutrient treatment as well as a cation channel inhibitor treatment (BaCl_2_) to directly test the sensitivity of the PET system, i.e. its ability to detect subtle differences between sodium transport rates within the same background plant species. Low-nutrient growth solutions have been shown to upregulate sodium transport in rice, the hypothesis being that nutrient deprivation, particularly of potassium, causes the plant to use similar ions, like sodium, to replace cellular roles [39, 40]. Similarly, inclusion of sodium channel blockers, such as BaCl_2,_ have been shown to have marked effects on decreasing sodium uptake in previous radiotracer experiments [16, 41–43]. In the experiments we report on herein, the differences created by the inhibitor and low nutrient treatments were accurately and reliably detected, indicating this system will have application in measuring subtle differences in sodium transport between treatments or genetic mutants.

The diurnal pattern of ^22^Na transport is an effect that has not previously been described experimentally, however has been hypothesized recently [44–46]. Future work is required to determine whether this fundamental sodium transport characteristic has important implications for how plants survive under conditions of elevated soil sodium conditions.

## Conclusion

The utility of clinical PET for multi-day continuous imaging of sodium transport in plants has been demonstrated using a ^22^Na tracer in barley plants, with four cases of varied nutrient and inhibitor levels imaged simultaneously. This technique provides a way to observe, in real time, genetic and treatment effects on sodium transport in plants, which will likely prove useful in the study of mutants lacking genes coding for various aspects of sodium transport at cellular and whole-plant levels.

## Methods

Plants were scanned continuously in a clinical PET/CT scanner (Siemens Biograph128 mCT), incorporating two diurnal and nocturnal periods, running for 65 h (Experiment 1) and 72 h (Experiment 2), respectively, for influx analysis.

### Plant material and growth conditions

Barley (*Hordeum vulgare* L. spp.* vulgare*—cultivar Bass) seeds were grown in a growth chamber (Conviron) at day/night conditions of 20 °C/15 °C, 12 h/8 h with a constant relative humidity of 60%, and a light intensity of 200 µmol/m^2^/s at plant height. Eight seeds per mesh collar were surface-sterilised in 1% (v/v) sodium hypochlorite for 15 min and washed four times with reverse-osmosis (RO) water for 15 min. Seeds were germinated in acid-washed sand for 3 days prior to transfer to 14-L hydroponic systems containing modified Johnson’s solution: solutions contained 0.5 mM Ca(NO_3_)_2_, 0.5 mM KH_2_PO_4_, 0.25 mM MgSO_4_, 0.25 mM K_2_SO_4_, 6.25 µM H_3_BO_3_, 20 µM FeEDTA, 0.5 µM ZnSO_4_, 0.5 µM MnSO_4_, 0.125 µM CuSO_4_, and 0.125 µM Na_2_MoO_4_ (pH adjusted to 6.3 to 6.35, using 1 M KOH). A total of 0.02 mM N was available in EDTA. Nutrient solutions were completely exchanged every 2 days to ensure depletion of nutrients did not exceed 15% of target concentrations.

At 9 days post germination, 50% of plants were switched to a ‘low nutrient’ solution (containing only 0.2 mM CaSO_4_, pH adjusted to 6.3 to 6.35 using 1 M Ca(OH)_2_) from the ‘high nutrient’ solution described above. At 11 days post germination, the plants were carefully moved, in nutrient solution systems, to the PET scanning facility for analysis.

### Plant preparation and scanning procedure

**Fig. 7:**
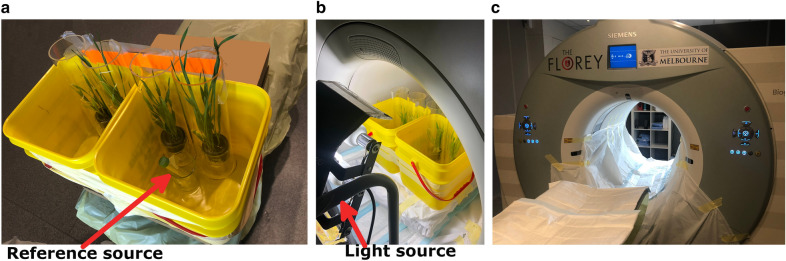
Experimental setup. **a** Four plant setups in two larger containers with the reference source, **b** Experimental setup in the scanner with the light source on, **c** Siemens Biograph128 mCT PET/CT scanner

Eight barley seedlings, affixed to ‘collars’, were placed in 150-mL beakers, two collars from each of the ‘high-nutrient’ and ‘low-nutrient’ hydroponic tanks. A cation-channel inhibitor, BaCl_2_, was added to one ‘low-nutrient’ and one ‘high-nutrient’ beaker, thereby creating four unique plant/nutrient solution combinations. Two beakers were placed in each of two larger plastic containers, maintaining equal distance between adjacent pairs. Each beaker was encircled by a PERSPEX^R^ cylinder to maintain radioactive isolation for each plant. A reference source with known activity was placed in a separate tube to check whether there was any drift in the scanner during the experiment. The containers were placed in the centre of the PET scanner field-of-view (Fig. 7).

The total quantity of ^22^Na was added to 12 mL of RO water and mixed thoroughly. Immediately prior to initiation of PET data collection, 3 mL of the ^22^Na radiotracer was administered into the solution in each beaker using a syringe with a 10-cm needle to completely mix the solutions and divide the total radioactivity as evenly as possible between the beakers (Table 2).

**Table 2:** Radioactivity levels and scan times

	High nutrient without BaCl_2_ radioactivity (MBq)	High nutrient with BaCl_2_ radioactivity (MBq)	Low nutrient without BaCl_2_ radioactivity (MBq)	Low nutrient with BaCl_2_ radioactivity (MBq)	Scanning times (hours)
Experiment 1	1.74	1.79	1.69	1.70	65
Experiment 2	2.25	2.09	2.00	2.03	72

In each experiment, CT images of the arrangement were acquired both at the beginning and at the end of the experiment with 120 kV tube voltage, and 313 mA and 243 mA tube currents in Experiments 1 and 2, respectively. The CT images were used in PET attenuation correction and for defining ROIs in the plants (see below). Following the initial CT scan, plants were imaged continuously in the PET detector ring with times as detailed in Table 2. Plants were illuminated by a white fluorescent light source with an intensity of 200 µmol/m^2^/s at plant height on a timer to provide the same 12 h day/8 h night light cycle to which the plants were originally entrained in the growth chambers.

### Image reconstruction

PET images were reconstructed on the Siemens scanner. Dynamic image sequences were reconstructed for each experiment, in 1-h intervals with 109 axial slices per time point, using the Point Spread Function/Time of Flight method with 1.59 mm × 1.59 mm in-plane voxel size, 2.027 mm slice thickness, scatter and decay corrections applied, in addition to attenuation correction using the CT images.

### Quantitative image analysis

The reconstructed PET images were analysed using MATLAB (Version R2019a) and ROIs in the CT images were delineated using the Athena DICOM viewer.

**Fig. 8:**
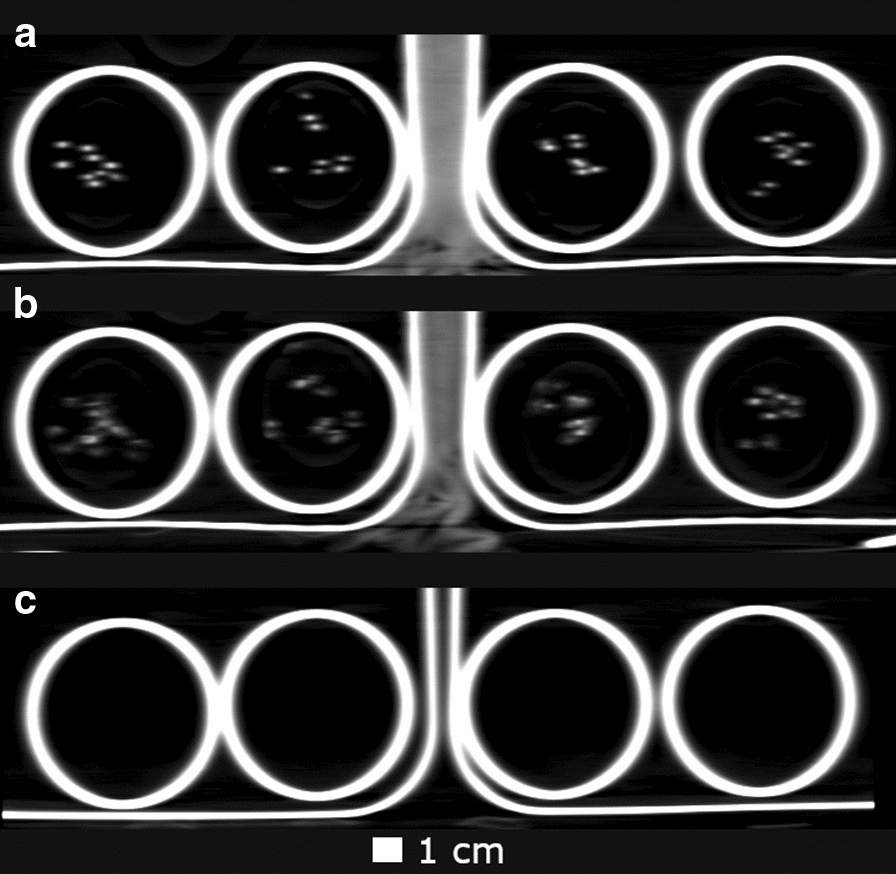
Coronal plane CT slices through **a** stems, **b** leaves, and **c** a plane above the level of leaves. Voxel size is 0.98 mm × 0.98 mm

**Fig. 9:**
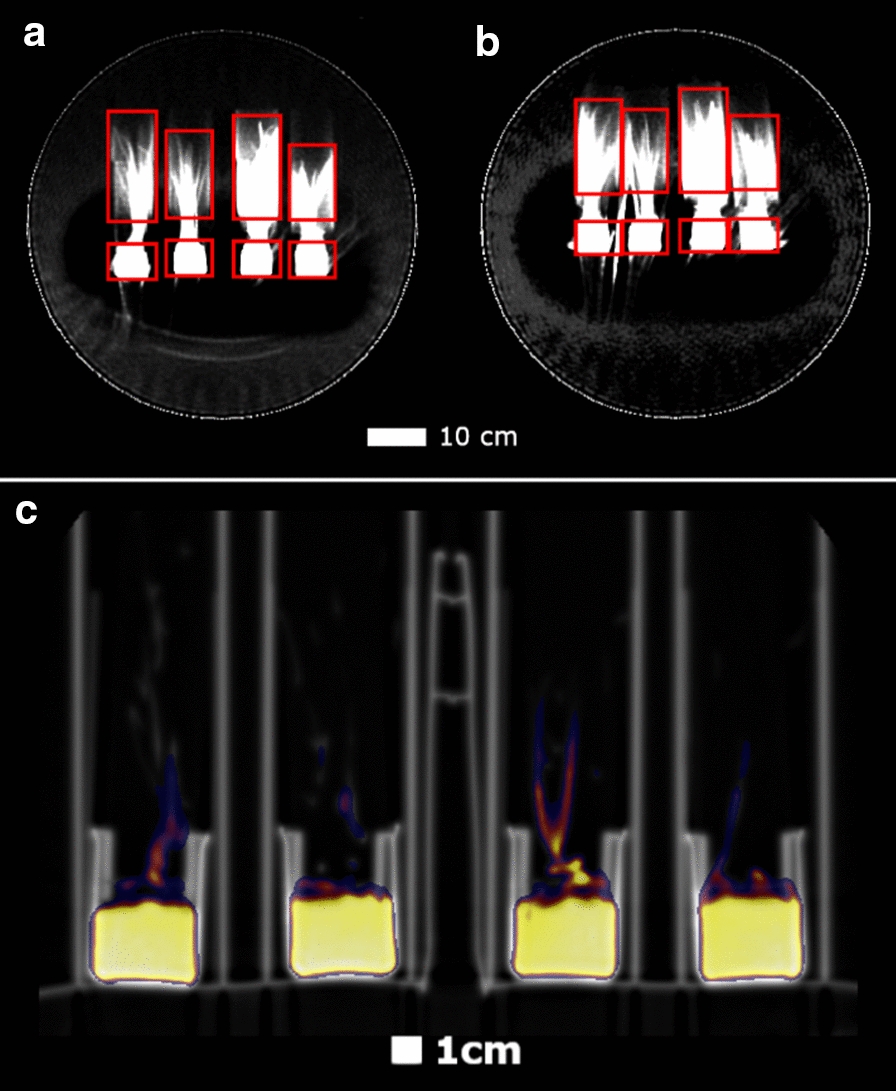
High contrast PET projection images with Regions of Interest overlaid (red boxes) for **a** Experiment 1, and **b** Experiment 2. Projections are given by the summation of all voxel intensities in the through-plane direction. The ROIs are 3D rectangular prisms, with identical spatial extent in the through-plane direction. **c** Axial PET image overlaid on CT image

As it was challenging to distinguish each plant’s anatomical boundaries precisely in the PET images, the boundaries, including roots, stem, and leaves, were identified in CT images. Across the experimental interval, the stems of the plants did not grow significantly, and the leaves grew marginally. Therefore, the CT images from the end of the experiment were used to notate heights of each region in the coronal plane. Figure 8 shows three different CT coronal plane slices in Experiment 2. The bottoms of the beakers were used as a reference and CT heights were converted to PET image space using voxel dimensions. The vertical boundaries of the PERSPEX^R^ cylinders were considered to be the vertical boundaries of each of the plants. 3D ROIs marked on PET images using this method are shown in Fig. 9a, b, with the PET images overlaid on CT in Fig. 9c. These projection images were generated by summing voxel values across slices (through-plane direction).

At each time point, radioactivity in each ROI was calculated by summing across the ROI’s voxels. To compare radioactivity between regions, values were normalized at each time point using the relevant plant’s total radioactivity, calculated at each time point. Rate of change of radioactivity over time was calculated using first central differences.

## Supplementary Information

**Additional file 1: Movie Exp 1.** Time-lapse video of ^22^Na dynamics for Experiment 1.

**Additional file 2: Movie Exp 2.** Time-lapse video of ^22^Na dynamics for Experiment 2.

## Data Availability

The datasets used and/or analysed during the current study are available from the corresponding author on reasonable request.

## Abbreviations

PET: Positron emission tomography
CT: Computed tomography
MRI: Magnetic resonance imaging
RO: Reverse osmosis
EDTA: Ethylenediamine tetraacetic acid
ROI: Region of interest
DICOM: Digital imaging and communications in medicine
FOV: Field-of-view
